# Reply to ‘Enhancing a phase measurement by sequentially probing a solid-state system'

**DOI:** 10.1038/ncomms11521

**Published:** 2016-05-09

**Authors:** Gang-Qin Liu, Yu-Ran Zhang, Yan-Chun Chang, Jie-Dong Yue, Heng Fan, Xin-Yu Pan

**Affiliations:** 1 Beijing National Laboratory for Condensed Matter Physics, Institute of Physics, Chinese Academy of Sciences, Beijing 100190, China; 2 Collaborative Innovation Center of Quantum Matter, Beijing 100190, China

Knott *et al*.^1^ argue in their comment on our recently published study^2^ that the enhancement of phase sensitivity is obtained by applying a phase shift twice, and thus not from the entanglement. We clarify that the entanglement between the two non-identical qubits is the crucial and decisive factor in the achieved enhancement, and the experimental technique used to encode the phases to qubits does not affect this conclusion.

By harnessing entanglement, quantum parameter estimation can yield higher statistical precision than purely classical approaches^3^,^4^,^5^. In our work^2^, two spins of different physical realization (an NV electron spin and its proximal ^13^C nuclear spin) are employed, and we demonstrate that their entangled state can give higher-phase statistical precision than their independent states. Phase information of spin qubits is of crucial importance because numerous external parameters, such as magnetic field^6^, electronic field^7^, temperature^8^ and pressure^9^, can be transferred into phase information and then measured with high sensitivity. The principle of quantum metrology works for all these external parameters, as it works for our encoded phases. Since the phases of microwave (MW) and radio frequency (RF) pulses are the controllable and measurable external parameters in our experiment, the claim that our scheme cannot measure an external parameter is not correct.

We first emphasize that entanglement of the two qubits is necessary to produce the enhancement of phase estimation in our experiment. Due to the energy structure of this four-level system, the used microwave pulse can only manipulate the state of electron spin, and the used radio frequency pulse can only manipulate the state of nuclear spin. This allows us to selectively encode phases to different target spins, simultaneously or in a sequential manner. As shown in Fig. 1a, one phase is encoded to the electron spin and another phase is encoded to the nuclear spin. If and only if the two qubits are entangled, their phases can be merged together and thus read out by one operation. For a comparison, as shown in Fig. 1b, we can prepare the two qubits to independent states, with the same phases. But in this case no quantum enhancement can be given; the employment of two qubits is equivalent to taking two repeat measurements on one qubit.

Also, we would like to claim that although the sequential pulses are employed to generate the entangled state, our experiment has significant differences from the sequential phase estimation scheme. As pointed in the comment and ref. 5, in such scheme, the *same* phase shifts are applied to the same superposition state, and no entanglement is needed (see Fig. 1c). In our entanglement-enhanced scheme, the two phases are encoded to qubits of different physical realization, by applying two completely different pulses (one is MW pulse, another is RF pulse). For a comparison, we also present a possible realization of the sequential strategy in our system (see Fig. 1c). In fact, sequential pulse is used to generate the entangled state of the hybrid quantum system, but it is not a necessary part of the phase-encoding process.

We then discuss the effect of qubit decoherence in our experiment. The thermal fluctuation of the surrounding ^13^C spin bath will bring random phases to spin qubits if they are under free evolution. The existence of qubit decoherence will weaken or even cancel the merits of entanglement^10^. However, in our protocol, the phases of spin qubits are encoded by MW or RF pulses, and the phase measurement is carried out right after the encoding. There is no free evolution between the state preparation and measurement operation. Meanwhile, the pulse duration is very short compared with the dephasing time of spin qubits. Therefore, the merits of maximum entangled state are well preserved and fully exploited in our experiment. We also discussed the decoherence behaviour of our system in the Methods section^2^.

We also wish to point out that the assumption of *ϕ*_MW_≠*ϕ*_RF_ (a special case is *ϕ*_MW_=−*ϕ*_RF_), as mentioned in Knott's comment, will break all quantum metrology strategies. If the entangled qubits have different phases, their measurement should most likely give ‘destructive interference' results. Meanwhile, the proposal Eq. (8) presented in the comment is not correct. If a single pulse with a frequency of RF+MW is applied to the system with an initial state of |0 ↑〉, no entanglement state can be generated because no transition in this system is resonant with this pulse.

In conclusion, we have shown that entanglement is necessary to our enhancement of phase estimation, and our work has significant difference with the sequential phase estimation scheme. In fact, the highlights of our work mainly lie in two aspects: (1) Our system has overcome the defects of postselection in the most common optical systems, which are fatal due to the fact that the measurement trials abandoned will eliminate the quantum-entanglement advantage over classical strategy. Our work in the solid system is a good complement for the previous experiments in optical systems. (2) We demonstrate a strategy with two entangled qubits of totally different physical realizations. This may enlighten further investigations on realizing quantum algorithms using different kinds of resources. Our results can benefit NV-based quantum metrology applications such as magnetometry^6^, thermometry^8^ and radio-frequency spectrum analysis^11^,^12^.

## Additional information

**How to cite this article:** Liu, G-Q. *et al*. Correspondence: Reply to ‘Enhancing a phase measurement by sequentially probing a solid-state system'. *Nat. Commun.* 7:11521 doi: 10.1038/ncomms11521 (2016).

## Figures and Tables

**Figure 1:**
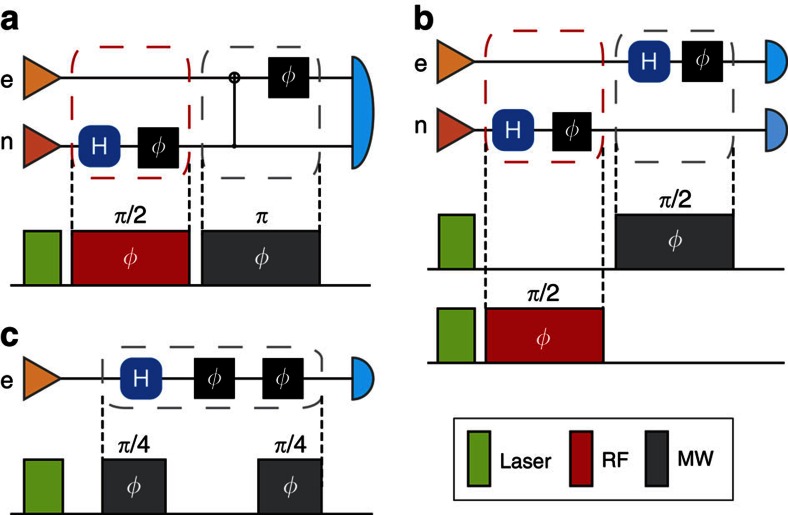
The quantum circuits and pulse sequences in our experiment. (**a**) The entanglement-enhanced strategy applied in our experiment. (**b**) The classical strategy applied in our experiment. (**c**) The possible sequential strategy in our set-up. The triangles on the left represent state preparation and the symbols on the right represent measurements. The grey boxes represent a unitary operation involving multiple probes^3^.
