# Erratum: Correspondence: Reply to ‘Enhancing a phase measurement by sequentially probing a solid-state system'

**DOI:** 10.1038/ncomms11865

**Published:** 2016-06-07

**Authors:** Liu Gang-Qin, Zhang Yu-Ran, Chang Yan-Chun, Yue Jie-Dong, Fan Heng, Pan Xin-Yu

Nature Communications 7: Article number: 11521 10.1038/ncomms11521 (2016); Published: 0592016; Updated: 06072016

The HTML version of this Correspondence was previously published with an incomplete title. This has now been corrected in the HTML; the PDF version of the paper was correct from the time of publication.

